# An evaluation of semi‐automated methods for collecting ecosystem‐level data in temperate marine systems

**DOI:** 10.1002/ece3.3041

**Published:** 2017-05-22

**Authors:** Kingsley J. Griffin, Luke H. Hedge, Manuel González‐Rivero, Ove I. Hoegh‐Guldberg, Emma L. Johnston

**Affiliations:** ^1^ Evolution and Ecology Research CentreSchool of Biological, Earth and Environmental SciencesUniversity of New South WalesSydneyNSWAustralia; ^2^ Global Change InstituteUniversity of QueenslandBrisbaneQLDAustralia

**Keywords:** kelp, marine survey, power analysis, spatial sampling, species distribution modeling

## Abstract

Historically, marine ecologists have lacked efficient tools that are capable of capturing detailed species distribution data over large areas. Emerging technologies such as high‐resolution imaging and associated machine‐learning image‐scoring software are providing new tools to map species over large areas in the ocean. Here, we combine a novel diver propulsion vehicle (DPV) imaging system with free‐to‐use machine‐learning software to semi‐automatically generate dense and widespread abundance records of a habitat‐forming algae over ~5,000 m^2^ of temperate reef. We employ replicable spatial techniques to test the effectiveness of traditional diver‐based sampling, and better understand the distribution and spatial arrangement of one key algal species. We found that the effectiveness of a traditional survey depended on the level of spatial structuring, and generally 10–20 transects (50 × 1 m) were required to obtain reliable results. This represents 2–20 times greater replication than have been collected in previous studies. Furthermore, we demonstrate the usefulness of fine‐resolution distribution modeling for understanding patterns in canopy algae cover at multiple spatial scales, and discuss applications to other marine habitats. Our analyses demonstrate that semi‐automated methods of data gathering and processing provide more accurate results than traditional methods for describing habitat structure at seascape scales, and therefore represent vastly improved techniques for understanding and managing marine seascapes.

## 1 INTRODUCTION

Healthy coastal and estuarine ecosystems have high social, economical, economic and environmental value, yet continue to suffer alarming degradation (Connell et al., 2008; Lotze et al., 2006). As a response, new management approaches such as coastal and marine spatial planning (CMSP) have received increasing attention in the past decade (Portman, 2016). Such approaches aim to integrate environmental, social and economic issues into policy generation (Portman, 2016). There are, however, few examples of coastal habitats with suitable data available to underpin such planning processes at relevant spatial and temporal scales, particularly in complex populated urban/industrial environments (Halpern et al., 2012). Faced with limited spatial and temporal replication, many CMSP projects rely on inadequate proxies, increasing the uncertainty in decision making, and reducing the ability of policy to detect and respond to environmental change (Foley et al., 2010; Smale, Burrows, Moore, O'Connor, & Hawkins, 2013). The paucity of data stems, in part, from the perception that the acquisition of ecological data will require substantial investments of time and money (Bates, Scott, Tobin, & Thompson, 2007).

There are a growing number of methods for efficiently collecting widespread benthic data, each with associated costs and limitations. Satellite and aerial photography, for example, are frequently used to cover large areas, but suffer limitations in resolution and water penetration (Kenny et al., 2003). These methods generally cannot achieve fine‐scale (<10 m) information about habitat condition or structure, especially in coastal waters subject to increased turbidity (Dekker et al., 2011). Other remote sensing technologies such as LiDAR (Zavalas, Ierodiaconou, Ryan, Rattray, & Monk, 2014), acoustic backscatter (Hill, Lucieer, Barrett, Anderson, & Williams, 2014), and hyperspectral imaging (Harvey, 2009) remain relatively inaccessible to many researchers. Most habitat mapping exercises now integrate remote sensing and field validation (Lyons, Phinn, & Roelfsema, 2011), yet maps used in marine policy and management are frequently restricted to broad, thematic classifications–an abstract representation of a highly heterogeneous reality (Holmes, Van Niel, Radford, Kendrick, & Grove, 2008). Coarse data may suit projects with broad aims, but without adequate biological understanding at a community (or finer) scale, a true ecosystem approach to managing marine systems remains unachievable (Hawkins, 2004). Historically, diver surveys have provided the most reliable means to overcome the limited ecological detail of large‐scale or remote sensing methods.

Diver surveys provide the highest taxonomic detail of all sampling methods, avoiding obstruction by canopy algae or topographic features, but are confined to small areas due to associated costs (Lindfield, Harvey, McIlwain, & Halford, 2014). To comprehensively and representatively sample a temperate reef, a diver survey would typically target a fixed number of quadrats and/or transects at each site, the exact position of which is either stratified to features of interest (e.g., Guidetti, Fraschetti, Terlizzi, & Boero, 2003) or randomly positioned. The total area sampled is the most important design factor in determining the precision and accuracy of a given estimate of subtidal organisms covering rocky reef (Drummond & Connell, 2005). The area sampled is typically a fixed variable across the sites, between <10 m^2^ (Edgar, Moverley, Barrett, Peters, & Reed, 1997; Oh, Edgar, Kirkpatrick, Stuart‐Smith, & Barrett, 2015; Wernberg, Kendrick, & Phillips, 2003) and 100's of m^2^ (Connell et al., 2008) depending on the sampling unit and level of replication. Data from these techniques are then analysed to contextualise variation, track change through time, or validate the direct comparison of multiple similar “reference” sites (Barrett, Buxton, & Edgar, 2009). Surveys of algae on temperate reefs have frequently described highly variable communities at all spatial scales, with communities on neighboring reefs often displaying markedly different spatial structures (Foster, 1990; Kennelly & Underwood, 1992).

A broad range of statistical literature has stressed the importance of assessing spatial patterns of variation, particularly when using a fixed sampling strategy across sites of markedly differing size or arrangement (Legendre et al., 2002). From an ecological perspective, variation in habitat structure has subsequent effects on species‐habitat associations (Gratwicke & Speight, 2005) and species richness (Griffin et al., 2009). Unfortunately pre‐existing knowledge about the layout of individual reefs, the patterns present in communities, or even the underlying geology, remains limited (Bennett et al., 2015). To avoid errors associated with site‐level variation in spatial autocorrelation (Diniz‐Filho, Bini, & Hawkins, 2003), hierarchical designs have been the backbone of marine ecology, whereby variance can be partitioned at multiple levels (Underwood, Chapman, & Connell, 2000). These designs held clear advantages for hypothesis testing, and alternative methods to investigate and account for variability across spatial scales (e.g., Stevens & Olsen, 2004) were inappropriate for large‐scale subtidal implementation due to a lack of efficient subtidal positioning systems. Today, we approach a technology‐driven paradigm shift in marine ecological data collection and analyses, but comparisons with traditional methods are lacking, especially in temperate marine systems (Ling et al., 2016; Perkins, Foster, Hill, & Barrett, 2016).

Technological developments in the last 2–3 decades have improved our ability to observe large areas of seabed in detail (Lambert et al., 2013), convert images into species data (Kohler & Gill, 2006), and analyse spatial patterns (Foster, Hosack, Hill, Barrett, & Lucieer, 2014). The use of images as a data source continues to grow in tandem with improvements in imaging systems (Beijbom et al., 2015). Software based around machine‐learning algorithms is now automating the conversion of images into data (Beijbom et al., 2015), reducing previously prohibitive processing time and costs (Foster, Harrold, & Hardin, 1991). Meanwhile, recent developments in positioning systems such as ultra‐short baseline (USBL) are making the collection of precisely geo‐referenced data feasible (Goldfarb, Wang, Bai, & Englot, 2015). High‐volume data collected by diver or vehicle‐mounted cameras and analysed with machine‐learning techniques will soon become commonplace, but there exists little detailed examination of the efficacy and appropriateness of this new data (Beijbom et al., 2015). One of the first examples of this “scaling‐up” of observations in marine systems demonstrated strong evidence to support the use of long (~2 km) photograph transects and automated image annotation software for change detection in coral reef communities (González‐Rivero et al., 2016). Our study deployed a DPV‐propelled camera system (González‐Rivero et al., 2014) to survey temperate reefs for the first time.

This study explores the use of a DPV‐driven imaging system (González‐Rivero et al., 2016) in temperate waters, comparing the automated classification of canopy algae in ~5,000 m^2^ of reef photographs with simulated conventional diver transect methods. Our study demonstrates the potential of large‐scale subtidal image surveys for efficiently capturing spatial variation in the abundance of a keystone species on temperate reefs. Our results validate investment of resources into these new sampling techniques, and emphasise the statistical value in geo‐referenced samples and observations. We assess differences in spatial heterogeneity between sites, and discuss the effect this variation may have on results from traditional survey designs. Furthermore, we demonstrate that simple environmental predictors (bathymetry, substrate data) and moderate amounts of occurrence data (*n* = ~200 quadrats) can produce valid distribution models for canopy algae, and spatial products of value to a wide range of fields.

## 2 METHODS

### 2.1 Regional context

The south‐eastern coast of Australia is typified by coastal estuaries supporting a diverse range of seagrass, reef, and soft sediment environments subject to variable water supply via the East Australia Current (Zann, 2000). Our study area, Sydney Harbour, is a modified system, where centuries of human development interact with a diverse rocky shoreline. The state government of New South Wales have regularly mapped key habitats in coastal estuaries including Sydney Harbour from aerial imagery (Creese, Glasby, West, & Gallen, 2009), but community scale (<5–10 m) data are lacking (Hedge et al., 2014; Johnston et al., 2015; Mayer‐Pinto, Underwood, & Marzinelli, 2015; Mayer‐Pinto, Johnston et al., 2015). Sydney Harbour has worldwide recognition as an iconic and biologically diverse estuary, but has typically been the subject of studies dissecting the effects of contaminants, invasive species, and artificial structures on marine ecology (Johnston et al., 2015). Remnant inundated and semi‐inundated habitats in Sydney Harbour include a variety of rocky reef habitats, seagrasses, soft sediments, saltmarshes, and mangroves, while more than half of the natural shoreline has been converted to hard artificial structures (Mayer‐Pinto, Johnston et al., 2015). This study focuses on rocky reef habitat and the Common Kelp *Ecklonia radiata* (C. Agardh) J. Agardh, a dominant canopy forming algae on reefs in Southern Australia (Bennett et al., 2015).

### 2.2 Data collection

In order to comprehensively and representatively sample temperate reefs nearby Sydney Harbour, sites of consistent 1–2 km long sections of reef habitat were identified from existing broad habitat maps (Creese et al., 2009). Benthic images were collected and analysed according to González‐Rivero et al. (2016), at depths of 2–12 m depending on reef topography. This system provided images collected every few seconds, while traveling, ensuring that overlap was avoided (mean distance between images was 3.8 m). Images were GPS located via towed buoy and taken at precise distances above the reef‐scape (as determined by the on‐board altimeter), to standardise the variation in width of observations by area surveyed. It should be noted that similar data could be produced by deploying standard digital cameras or underwater vehicles, but that apart from rapidly capturing high‐quality imagery over large areas (González‐Rivero et al., 2014), this system allowed safe access to areas with unknown or complex topology, shallow water, and high wave action, which may be considered of risk to more expensive or complex remote tools.

**Figure 1:**
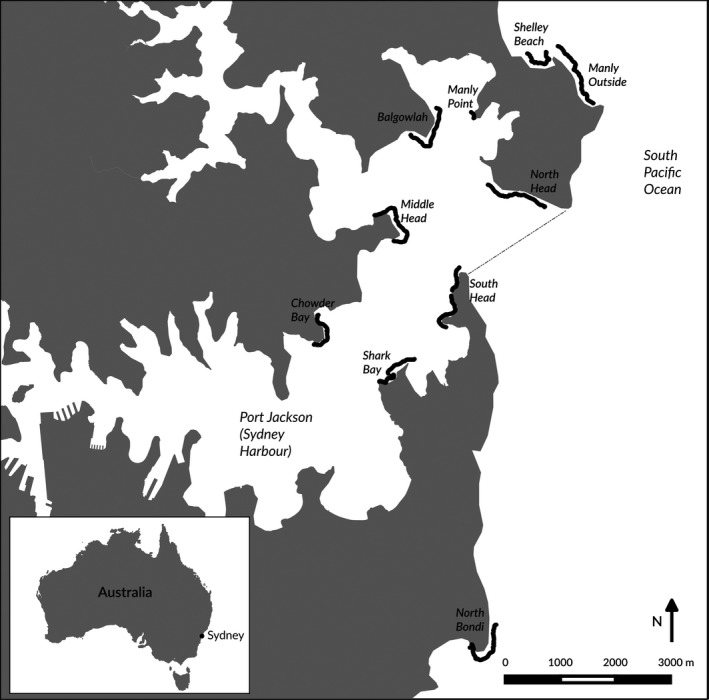
GPS tracks of image surveys at reef sites around Sydney Harbour (black)

The complete seabed image survey produced 11,151 geo‐spatially referenced records of Common Kelp *E. radiata* percentage cover from 1 × 1 m photograph quadrats spread between 10 sites around Sydney Harbour. Of the surveyed sites, three were outside Sydney Harbour (Manly Outer, Shelley Beach, and North Bondi). The remaining seven sites (North Head, South Head, Manly Point, Balgowlah, Middle Head, Nielsen Park and Chowder Bay; Figure 1) were located within the Harbour. The two smallest sites (Shelley Beach and Manly Point) were excluded from the following statistical analyses, as they provided insufficient continuous sections of reef.

### 2.3 Data preparation

Once extracted, downward‐facing seabed images were corrected for fisheye lens distortion and color balanced with Adobe Bridge (Ver. 6.1.0.115) and Photoshop (ver. 15.1.0) using the standard automatic color balance tool and in‐built lens correction plugin. The images were programmatically cropped to a standard 1 × 1 m photo quadrat size following calibrated trigonometric formulas calculated for the “XL Catlin Seaview Survey” “Global Reef Record”(González‐Rivero et al., 2014). Depending on the camera altitude, up to 16 quadrats each of 1 m^2^ were cropped from the images, avoiding image edges (subject to distortion) and without overlapping. Images which were likely to be distorted due to close (<0.4 m) or distant (>5 m) proximity to the seabed were excluded from further analysis programmatically. The few remaining blurred or unsuitable images were discarded during thorough visual checks. Following these quality control criteria resulted in 5,880 valid image quadrats, distributed across the 10 sites at an even density, with the final number of images at each site dependent on the extent of reef present.

### 2.4 Biotic classification

The image quadrats and metadata were then uploaded to a software tool (CoralNET) for supervised classification of biota. The CoralNET software is explained in detail in (Beijbom et al., 2015), and works in a similar manner to other image classification procedures such as “Coral Point Count (CPC)”(Kohler & Gill, 2006). In this case, biota were annotated at 25 randomly overlaid points, following the Australian morphology‐based CATAMI classification scheme, which aims to standardise the classification “tags” used on benthic images (Althaus et al., 2015). For analysis of the effect of photo quadrat dimensions, number of points digitised, and subsampling strategies, refer to Perkins et al. (2016). Where possible, biota were further classified to genus or species. As kelp *E. radiata* is the major canopy forming algae on reefs in the region, and less subject to seasonal variation than other canopy algae groups (Kennelly & Underwood, 1992), this was chosen as the focal species. A single operator was used during classification to minimise error associated with multiple observers (Beijbom et al., 2015). Ultimately, 21.21% of classification was completed manually, which resulted in the CoralNet software identifying kelp *E. radiata* with ~93% accuracy (calculated by comparing a subset of training data (expected results) with predictions, both in the form of standard accuracy and Cohen's kappa (Beijbom et al., 2015). The effect of accepting these automated classifications with inherent error has been shown to be comparable to error introduced by human error/bias (Beijbom et al., 2015). The combined cover data, generated both manually and by CoralNET (a semi‐automated approach), were then accepted as the species abundance database for the following analyses.

### 2.5 Assessment of simulated traditional survey designs

**Figure 2:**
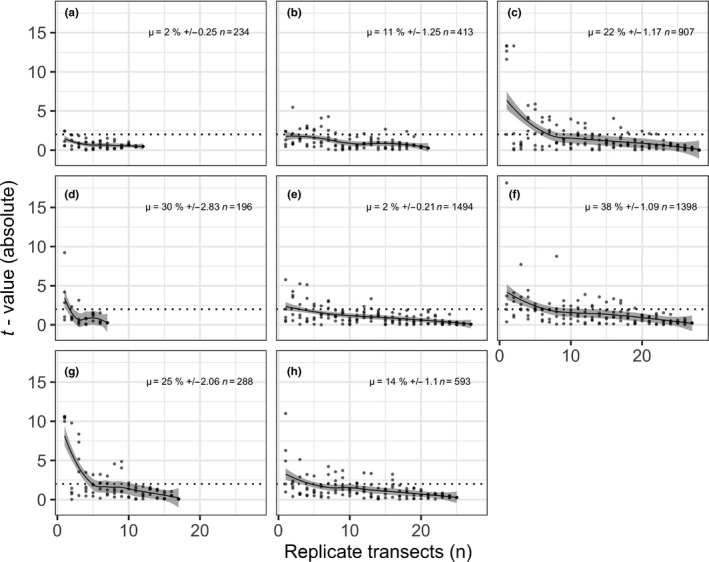
The difference (absolute *t*‐value) between “true” kelp cover at a site (from the entire image survey), and kelp cover from (*n*) randomly selected 50 m “transect” sections, with transect selection repeated 5 times with replacement. Points are slightly transparent to allow for interpretation of overplotting. A LOESS smoother (gray) with 95% confidence interval, and a dotted line at t = 2 is included to aid interpretation. Mean kelp cover, standard error (2 d.p.), and *n* quadrats from complete image surveys are displayed in the upper right of each frame. Sites: (a) Chowder Bay; (b) Balgowlah; (c) Manly Outside; (d) Shark Bay; (e) North Head; (f) North Bondi (g) South Head; and (h) Middle Head

In this simulation, we used the kelp cover data from all photo quadrats as the conceptual “true” value of kelp at each site. To synthesise data from traditional diver surveys, we randomly extracted 50 × 1 m transects from the complete survey at each site without replacement. To accomplish this, the distance between each photograph point was calculated from the GPS positions, and images in 50 m contiguous sections isolated in R. This procedure was repeated 5 times at each site to simulate the effect of random transect placement. Differences between the “true” kelp cover and the simulated transects were then compared using *t*‐values (Figure 2).

We then employed variography to assess variation in community spatial structure at the study sites, and investigate the influence of spatial heterogeneity on the simulated traditional surveys. Spatial neighbors were mapped and Moran's *I* correlation coefficient calculated to describe the level of statistical similarity between kelp cover measurements separated by a given distance. “Null” correlation was assessed by generating random cover values at the same locations.

### 2.6 Spatial analysis

We used Species Distribution Modelling (SDM) as a tool to map kelp *E. radiata* over whole regions, at a fine spatial resolution. We modeled the probability of kelp cover over the Sydney Harbour study sites as a function of latitude (LAT), longitude (LONG), bathymetry (BATH), and the extent of viable rocky substrate (RREEF; from existing qualitative habitat maps; Creese et al., 2009). These variables were selected as they are of biological significance: depth gradients are well‐described in coastal ecosystems, and kelp *E. radiata* requires rocky substrate for establishment. Latitude and longitude can be proxies for spatial gradients in a multitude of biotic and abiotic factors. A final consideration was the broad availability of these predictors for the majority of Australian coastal waters.

The SDM was trained using the relative abundance data from our image classification. This strategy was chosen in preference to presence‐only or presence/absence SDM, as abundance data improves the effectiveness of SDM, especially for widespread species (Howard, Stephens, Pearce‐Higgins, Gregory, & Willis, 2014). We implemented a generalised linear model (GLM) with a binomial family, such that: *p* = (successes, number of trials [25]) ~ LATITUDE + LONGITUDE + BATHYMETRY + REEF. Following standard SDM techniques (Elith & Leathwick, 2009), we then predicted kelp cover onto a 5 m resolution raster grid of our study area in Sydney Harbour (Figure 4). This resolution was chosen based on the availability of environmental data.

### 2.7 Predictive accuracy

Leave‐one‐out (LOOCV) and *k*‐fold (*k* = 10) area under the receiver operating curve (AUC) cross‐validation strategies were used to assess the predictive accuracy of the distribution model described above. Furthermore, to understand the efficiency of our methods, we were interested in the minimum number of quadrats needed to optimise our SDM. SDMs of the above equation were trained with an increasing number of randomly selected quadrats (repeated 5 times/*n*) and validated using the LOOCV approach until there was no improvement in accuracy. It should be noted that the best practice for the cross‐validation of SDM continues to be debated, but LOOCV and AUC are most widely used.

## 3 RESULTS

Mean kelp cover ranged from 2% to 38% between the study sites (Figure 2). Shark Bay had the highest relative kelp abundance of sites in the harbor (30%), while kelp was in low abundance at Chowder Bay and North Head (~2%). Outside of the harbor, quadrats at Manly had 22% mean kelp cover, and North Bondi had the highest cover of surveyed sites (38%).

Kelp cover data were markedly different to “true” (whole site) data under simulations with low *n* (<10) replicate transects (Figure 2). This was particularly evident at North Bondi (F, Figure 2). Increasing the replication to ~10 transects produced more consistent and similar results to “true” values at Balgowlah (B), Manly Outside (C), North Head (E), South Head (G), and Middle Head (H) (Figure 2). The simulation suggests that upwards of 20 transects would be required to produce repeatable, similar results at North Bondi (F).

**Figure 3:**
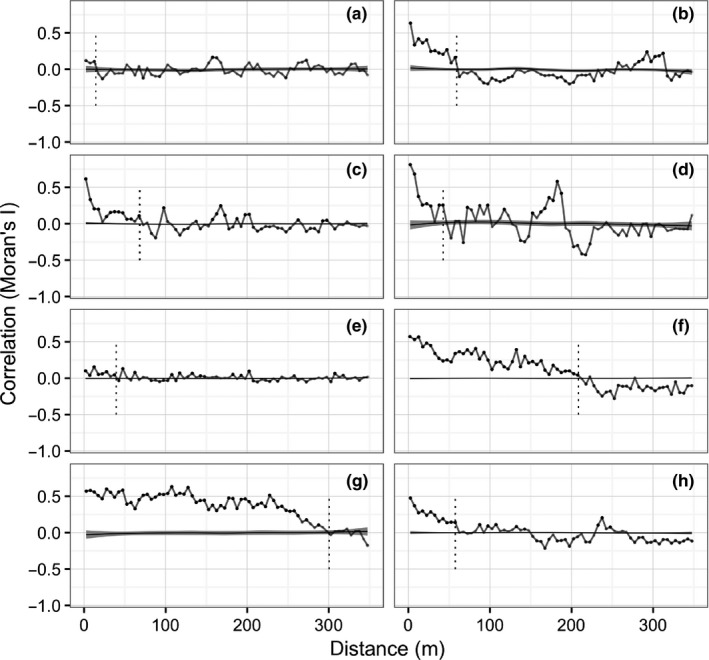
Omnidirectional correlogram of kelp *Ecklonia radiata* cover at each site. The correlation index used is Moran's *I*, at a given distance (m) between samples A LOESS smoother (gray) and 95% confidence interval is displayed for null (random) correlation at the same locations. The approximate x‐intercept is demarcated with vertical dotted line. Sites: (a) Chowder Bay; (b) Balgowlah; (c) Manly Outside; (d) Shark Bay; (e) North Head; (f) North Bondi (g) South Head; and (h) Middle Head

Spatial correlation was weak (*I* < .25) at Chowder Bay and North Head, suggesting that kelp abundance was randomly organised, rather than forming distinct patches (Figure 3). At the other sites, kelp abundance was strongly autocorrelated (*I* > .5) between nearby samples, and showed at least moderate correlation (*I* > .25) over 30–80 m, indicating that kelp abundance was arranged in distinct heterogeneous patches.

**Figure 4:**
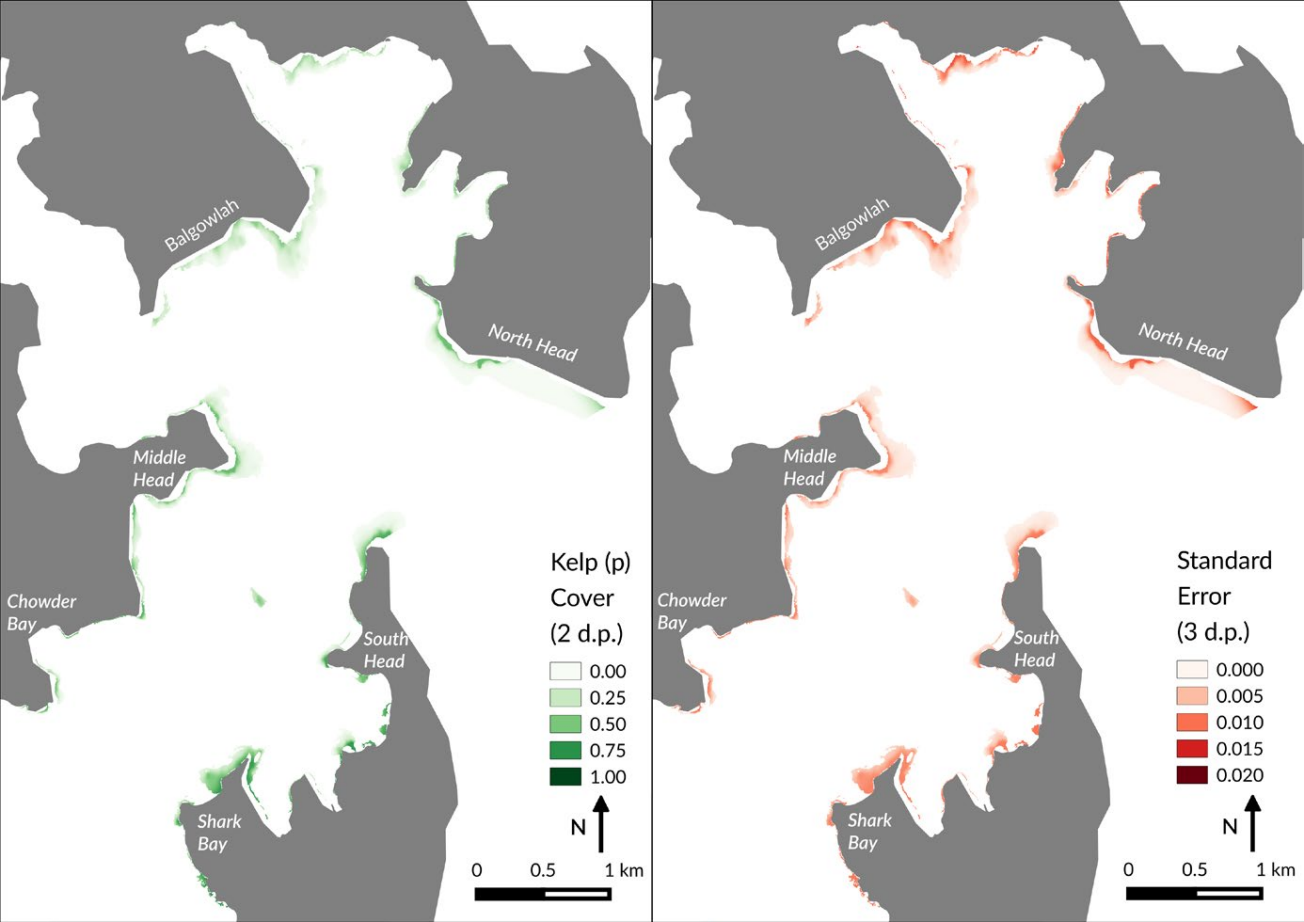
Predicted cover (left pane) and standard error (right pane) of kelp *Ecklonia radiata* across reefs within the sampling area in Sydney Harbour. This prediction can be considered as relative abundance within a given area. Mean AUC = .71; estimated prediction error = .043, .043 (see Appendix S1)

There were no noticeable broad spatial trends in the SDM predictions, although predicted *E. radiata* was generally higher in areas closer to the shore (Figure 4). Shark Bay was predicted to have a visibly larger area of kelp cover than other sites of a similar size. Predicted kelp at North Head was highly restricted and mostly absent toward the harbor entrance.

**Figure 5:**
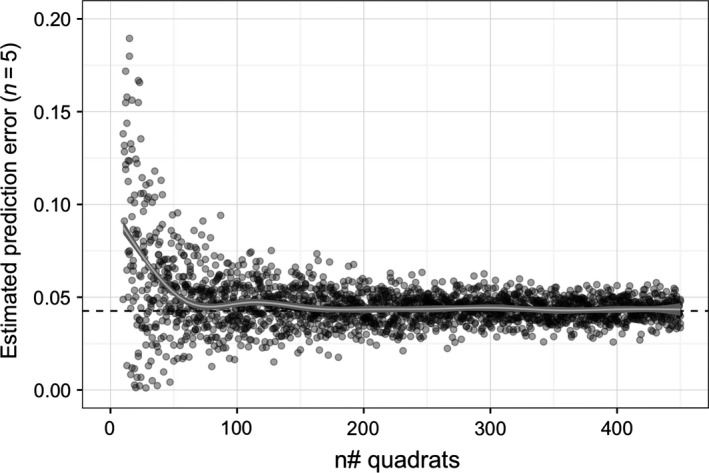
Effect of varying training data volume on leaves one out cross‐validation of GLM prediction error (*n *= 5). Points are transparent to aid visualisation of overplotting, and the plot is restricted to show the effect of varying the inclusion of 10–450 training data points (quadrats). A LOESS smoother (white line) and 95% confidence interval (gray ribbon) are displayed along with the estimated (LOOCV) prediction error from the complete SDM (~3,200 training points; dashed line)

The estimated prediction error from “leave‐one‐out” cross‐validation reached a visual asymptote after ~200 training points (Figure 5). This result indicates that the GLM fitting kelp cover to bathymetry, latitude, longitude, and reef boundaries across the study area could potentially be reliably trained with substantially less data than we collected.

## 4 DISCUSSION

Our semi‐automated subtidal survey generated the first comprehensive records of kelp cover in Sydney Harbour, covering over ~5,000 m^2^ of temperate reef. We found that *E. radiata* distribution patterns were driven by site‐level effects, and an unexpected, negative relationship with depth. Using this data to simulate a range of traditional diver‐based sampling designs, we found the effectiveness of a traditional survey would be highly dependent on the level of spatial structuring in our habitat‐forming species. Simulation of poorly replicated (<100's m^2^) survey designs frequently produced spurious results. Our semi‐automated methods of data gathering and processing were simple and inexpensive to deploy, and represent a viable alternative to traditional surveys, for collecting detailed information on habitat structure at seascape scales. Coupling our photographic surveys with Species Distribution Modelling (SDM) produced broadly useful maps with the first fine‐scale information about kelp cover over temperate reefs in Sydney Harbour. Our results emphasise the value of scaling‐up observations in temperate reef systems, especially when site or region level trends are of interest.

### 4.1 Evolving metholodologies for the study of marine habitats

New technology is enabling a transition toward spatial analyses and model‐based means of studying marine habitats. Challenges associated with positioning samples, combined with a lack of readily available environmental data, have caused a delay in the uptake of spatial analyses in marine systems compared with terrestrial counterparts. Today however, open‐access environmental data, subsea positioning solutions (e g., USBL systems), and strategies for targeted collection of samples (Stevens & Olsen, 2004) are making spatially driven approaches more feasible (Foster et al., 2014). The integration of detailed field samples and broader‐scale observations from remote sensing has, for example, enabled previously unattainable, detailed mapping of seagrass habitat over large spatial scales (Roelfsema, Lyons, Dunbabin, Kovacs, & Phinn, 2015). Automated underwater vehicles (AUV's) are now providing large datasets requiring careful consideration (Perkins et al., 2016). These new tools are helping us access difficult to reach reefs, improving our ability to understand the influence of key grazers (Ling et al., 2016) and measure metrics of reef ecology at a continental scale (Marzinelli et al., 2015). In this study, we developed a semi‐automated approach for examining fine‐scale patterns of variability in a dominant habitat‐forming alga on the temperate reefs of a complex urbanised estuary.

Kelp is a charismatic, dominant, habitat‐forming species on temperate reefs. Here, within‐site variability helped us to evaluate how high‐performance survey technologies might contribute to overcoming the limitations of traditional surveys to best represent canopy algal distribution, and make generalisations about their ecology. Before recent technological developments, it was unfeasible to saturate a subtidal study site with samples or accurately locate measurements underwater for spatial analysis (Foster et al., 2014). In this study our sites displayed a range of spatial structures, including sites with correlation at all distances and sites exhibiting spatial randomness. This is unsurprising; nearby reef sites often display stark differences in physical arrangement (Tuya, Wernberg, & Thomsen, 2008), key biotic processes (Andrew, 1993), and community spatial structure (Wernberg et al., 2003). As a result of this site‐level variation, traditional style studies using small sampling units and fixed replicates across varying sites are known to carry increased type I error rates (Legendre et al., 2002). Effect size has been shown to change substantially once spatial structures are accounted for (Diniz‐Filho et al., 2003). Despite this knowledge, the traditional reef survey design, using only three to four replicates per site, remains archetypal. In the present study, synthesised surveys using three to four replicate 50 × 1 m transects produced unreliable estimates of canopy algal cover compared with higher‐replication designs. New technology‐ and model‐driven approaches are predicted to dramatically increase the volume, and spatial footprint of data collected and analysed across our oceans and estuaries.

It should not be assumed that the use of high technology and contemporary model based analyses will result in greater costs. Here, we demonstrated that spatial analyses similar to the more sophisticated sampling programs mentioned above are both worthwhile and achievable using every‐day tools. We used inexpensive (<$10K AUD) off‐the‐shelf equipment and free open‐source software such as R for most analyses. We demonstrate that the collection of images and classification with machine‐learning software can efficiently produce large sets of species occurrence data. Furthermore, our spatial modeling framework used readily available environmental predictors, and we demonstrated that reliable distribution models could be produced with far fewer data points than we collected.

### 4.2 Kelp distribution on an urbanised coast

Rather than elucidating broad, estuary‐scale trends, our observation and SDM predictions indicate that kelp distribution in Sydney Harbour is relatively site‐specific. The distance from the open coast did not appear to drive the distribution of kelp in our study area. For example, kelp cover at Chowder Bay, within the harbor, was more similar to the most open/exposed site (North Head) than the opposite side of the harbor (Shark Bay). Across most sites, depth was a major factor determining kelp distribution. In open coast exposed systems, *E. radiata* remains common below depths of 20 m (Bennett et al., 2015), but at the sites within Sydney Harbour our observation and SDM predictions was that kelp is restricted to shallow areas of <10 m depth (see Appendix S1). Exposure to waves and water movement can influence kelp structure (Fowler‐Walker, Wernberg, & Connell, 2005) and community processes (Wernberg & Connell, 2008), which may contribute to the shallow limit of kelp survival within the sheltered harbor. It is likely that increased turbidity within the estuary and harbor also reduces the light attenuation depth (Mayer‐Pinto, Johnston et al., 2015), in turn limiting kelp growth.

Given the apparent lack of regional trends, it is likely that reefs in Sydney Harbour are subject to local‐scale effects of interacting stressors such as water quality and grazing herbivores, which our simplified model could not explicitly test. On reefs in this region, urchins can graze down more than 50% of canopy algae, even creating canopy‐free “barrens” (Andrew, 1993), and poor water quality facilitates dominance by low‐functioning turfing alga (Gorgula & Connell, 2004). The synergistic interaction of these stressors can drive the reduction of kelp‐covered reef, toward a stable and functionally inferior turf‐dominated state (Russell & Connell, 2005). While we have demonstrated the usefulness of a fine‐scale spatial modeling approach, a more complex methodology would be required to adequately incorporate potential interactions between kelp cover, urchin grazers, and water quality. Observations from our photographs, however, suggest that reef composition in Sydney Harbour is driven by the interaction of such processes at a local scale. For example, the community composition at North Head and Chowder Bay was indicative of acutely different stressors. From our observations, crustose coralline algae dominated the clear space between stands of kelp at North Head (K. J. Griffin, unpublished data), which is typical of urchin‐driven barren reefs on exposed open coast in the region (Andrew, 1993). Canopy‐free areas of reef at Chowder Bay, however, were dominated by turfing algae (K. J. Griffin, unpublished data), similar to disturbed reefs near other Australian coastal cities (Connell et al., 2008). While we cannot assume causality from this trend, the fine scale at which we can make observations highlights the value of both upscaling data collection, and using spatial analysis for both purely ecological and applied purposes (e.g., spatial planning).

### 4.3 Applications for “new” tools

Tools such as we have demonstrated here may improve our ability to monitor coastal ecosystems, offering new insights into health and function over large areas. Ultimately, these tools will enhance management frameworks via real‐world overlaps in both human and ecological data (Halpern et al., 2012). Coastal habitats are under increasing threat from local and global stressors, yet are amongst the most economically valuable ecosystems (Costanza et al., 1997). Worldwide, we have seen sudden, and often unexpected losses of key habitat‐forming species in the coastal zone. These events have vastly improved our understanding of the sensitivity of coastal environments to seemingly small disruptions in key processes (Barbier et al., 2008). The terms “ecological threshold,” “critical transition,” and “tipping point” explain this transition of a species or community past a point of no return (Barnosky et al., 2012). Contemporary CMSP initiatives now employ guidelines around “trigger‐points,” and integrate “adaptive” or “responsive” strategies, to watch and act before losses are incurred (Douvere & Ehler, 2010). Experiments have demonstrated that “early warning” indicators can be detected before these critical transitions, but these indicators are largely unknown from natural systems (Dakos et al., 2012). In part, the lack of knowledge around warning signals could be due to the paucity of comprehensive (long term, fine spatial, and temporally replicated) monitoring data in coastal systems (Bates et al., 2007). Management initiatives employing these central concepts (“adaptability” and “responsiveness”) will, by necessity, require tools with the ability to both cover broad spatial areas and detect these potentially subtle ecological indicators (Mills et al., 2015).

The marine monitoring programs of the future will respond to the need for more and better data by nesting sampling tools based on their sensitivity (Van Rein, Brown, Quinn, & Breen, 2009), and employing more sophisticated sampling designs (Foster et al., 2014). In the case of our study, the rapid generation of widespread and dense species records from photographic surveys gave clear advantages over more traditional methods. Similar results could be obtained with simpler high‐resolution underwater cameras, or more complex remote imaging systems. Regardless of the capture method, nesting in situ image surveys within a broader monitoring strategy could mean deploying long photograph‐based surveys at strategic locations to provide high detail, while relying on coarser methods (e.g., camera drops, aerial imaging, and remote sensing) elsewhere (Lyons et al., 2011).

While the advantages of these technologies are expected to be applicable to a range of species and habitats (e.g., González‐Rivero et al. (2014)), it is clear that the sampling strategy must adapt as usual, to the specific goals. Rare or patchily distributed species will likely require careful survey design, and photographic methods which remain above canopy level may be inappropriate (Perkins et al., 2016). There are likely to be other effects on algal health such as bleaching or disease, which are not immediately detected from automatically classified photographs (Campbell, Harder, Nielsen, Kjelleberg, & Steinberg, 2011). It is unlikely that traditional visual survey techniques would uncover these effects more readily. Instead, spatially balanced (Stevens & Olsen, 2004), or targeted designs could be implemented from SDM outputs, to focus on relevant seabed or habitat features.

## 5 CONCLUSION

Our study highlights the opportunities presented by new technologies for upscaling our observations and understanding of subtidal ecosystems. Our results suggest that traditional methods may be ineffective at capturing reliable estimates of canopy algal cover. The assumption that contemporary spatial analyses require substantial investment in technology, and larger datasets are likely a fallacy–on the contrary, these methods are rapidly becoming cheaper and more accessible. Ultimately, the best sampling method will be task specific, but the most significant aspects of a modern sampling tool should be to quickly cover large areas, and collect GPS locations for each record. From the resulting widespread species records, spatial analyses such as SDM can, with relative ease, improve our understanding of fine‐scale patterns over temperate reefs. This result should be of particular significance to those designing field programs to select reference sites, track change in communities at a variety of sites through time, or recommend best practice for management actions in marine systems.

## 6 DATA ACCESSIBILITY

The data described within will be publicly available at the time of publication, via Temperate Reef Base. R code necessary to reproduce analyses will be available at GitHub/KingsleyG. Full model specifications can be found in Appendix S1. R packages used included as follows: knitr (ver. 1.15.1 (Xie, 2013)), dplyr (ver. 0.5.0) and stringr (ver. 1.2.0) (H. Wickham, 2014), sp (ver. 1.2‐4; Pebesma et al., 2015), raster (Hijmans et al., 2015), dismo (Hijmans, Phillips, & Elith, 2016), spdep (ver. 0.6‐11) (Bivand, 2015), biOps (ver. (M. Bordese, 2013)), and boot (ver.1.3‐18).

## CONFLICT OF INTEREST

None declared.
