# Supplementary material for: An evaluation of semi‐automated methods for collecting ecosystem‐level data in temperate marine systems
Source: Ecol Evol. 2017 May 22;7(13):4640–50. doi: 10.1002/ece3.3041 (PMC5496513; doi:10.1002/ece3.3041)

Supplementary Materials A

All code and data neccesary to reproduce the analysis undertaken in this publication is available via Github and Temperate Reef Base

Spatial Data Sources

The code necessary for coercing the model predictors (bathymetry, viable substrate) into spatial form is available in the script `R/SDM_predictors_5m.R`.

Coastlines

The coastline used throughout this project was the Geoscience Australia '100k coastline' and accompanying shapefiles of coastlines for estuaries within Australia. Both the continent-wide and estuary coastlines are available online via Geoscience Australia.

Bathymetry

Bathymetry data was made available by Sydney Ports Corporation, and the NSW Office of Environment and Heritage. The data was generally just transformed into a mean depth per pixel raster. In areas of the study area where depth data was sparse, records were interpolated via thin-plate spline regression.

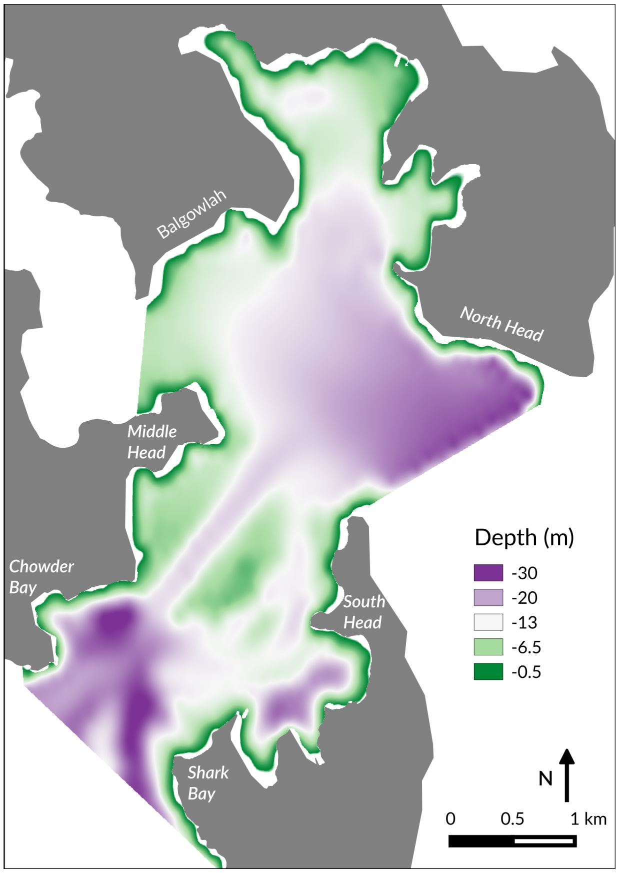

Viable Substrate

Existing habitat and substrate mapping supplied the information neccesary to create rasters of binomial presence or absence of rocky substrate necessary for the growth of our focal species. The original data was merely transformed into a raster format and clipped to our project area.

Latitude and Longitude

Latitude and longitude rasters were generated from the centroids (x, y) of each cell.

Covariance

The model covariates displayed the following pairwise correlation:

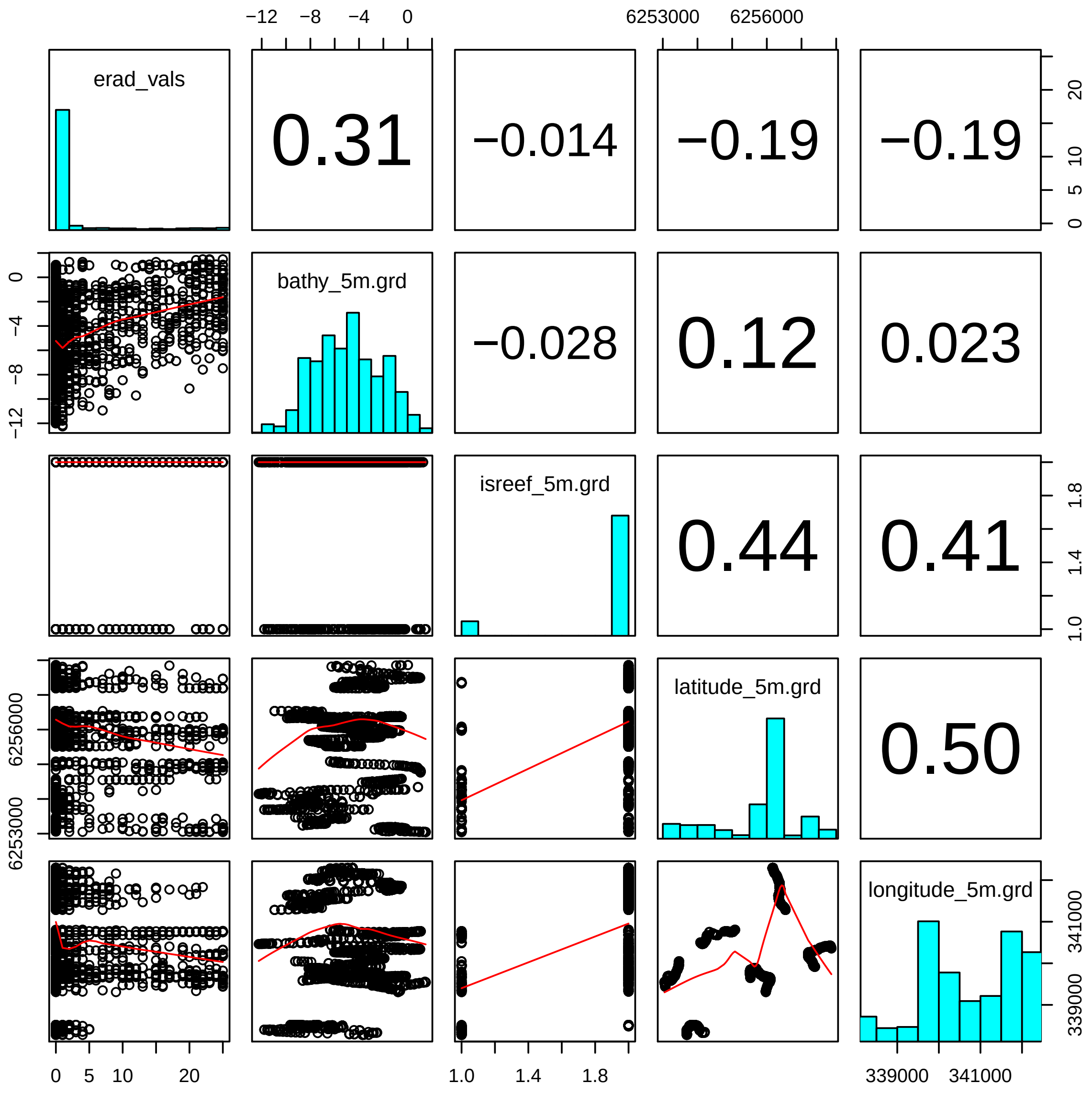

Model selection

The code necessary to reproduce the modelling is available in the script `R/SDM_Kelp_5m.R`. The binomial glm was specified with the following results and post-hoc model selection results:

```
##  [1] ""
##  [2] "Call:"
##  [3] "glm(formula = cbind(erad_vals, 25 - erad_vals) ~ isreef_5m.grd + "
##  [4] "    bathy_5m.grd + latitude_5m.grd + longitude_5m.grd, family = binomial, "
##  [5] "    data = sdmdata)"
##  [6] ""
##  [7] "Deviance Residuals: "
##  [8] "    Min       1Q   Median       3Q      Max  "
##  [9] "-7.5889  -2.0199  -1.2640  -0.7187  12.9205  "
## [10] ""
## [11] "Coefficients:"
## [12] "                   Estimate Std. Error z value Pr(>|z|)    "
## [13] "(Intercept)       2.172e+03  6.934e+01   31.33   <2e-16 ***"
## [14] "isreef_5m.grd1    1.047e+00  4.309e-02   24.30   <2e-16 ***"
## [15] "bathy_5m.grd      3.567e-01  5.159e-03   69.15   <2e-16 ***"
## [16] "latitude_5m.grd  -3.208e-04  1.137e-05  -28.23   <2e-16 ***"
## [17] "longitude_5m.grd -4.905e-04  1.458e-05  -33.65   <2e-16 ***"
## [18] "---"
## [19] "Signif. codes:  0 ‘***’ 0.001 ‘**’ 0.01 ‘*’ 0.05 ‘.’ 0.1 ‘ ’ 1"
## [20] ""
## [21] "(Dispersion parameter for binomial family taken to be 1)"
## [22] ""
## [23] "    Null deviance: 33877  on 3217  degrees of freedom"
## [24] "Residual deviance: 25478  on 3213  degrees of freedom"
## [25] "AIC: 27705"
## [26] ""
## [27] "Number of Fisher Scoring iterations: 5"
## [28] ""
## [29] "Single term deletions"
## [30] ""
## [31] "Model:"
## [32] "cbind(erad_vals, 25 - erad_vals) ~ isreef_5m.grd + bathy_5m.grd + "
## [33] "    latitude_5m.grd + longitude_5m.grd"
## [34] "                 Df Deviance   AIC"
## [35] "<none>                 25478 27705"
## [36] "isreef_5m.grd     1    26150 28374"
## [37] "bathy_5m.grd      1    31257 33482"
## [38] "latitude_5m.grd   1    26307 28531"
## [39] "longitude_5m.grd  1    26674 28899"
```

The model terms were all found to be significant in explaining kelp cover, and post-hoc selection demonstrated that each of the covariates substantially contributed to the model fit (and thus were retained).

Diagnostics

As a generalised linear model of binomial family, we expect to see structures in the model summary plot (residuals vs. fitted values; scale-location; normal q-q, residuals vs. leverages), which reflect the generalised aspects of the model (not to be confused with the relative lack of structures in diagnostics plots expected for "true" linear models).

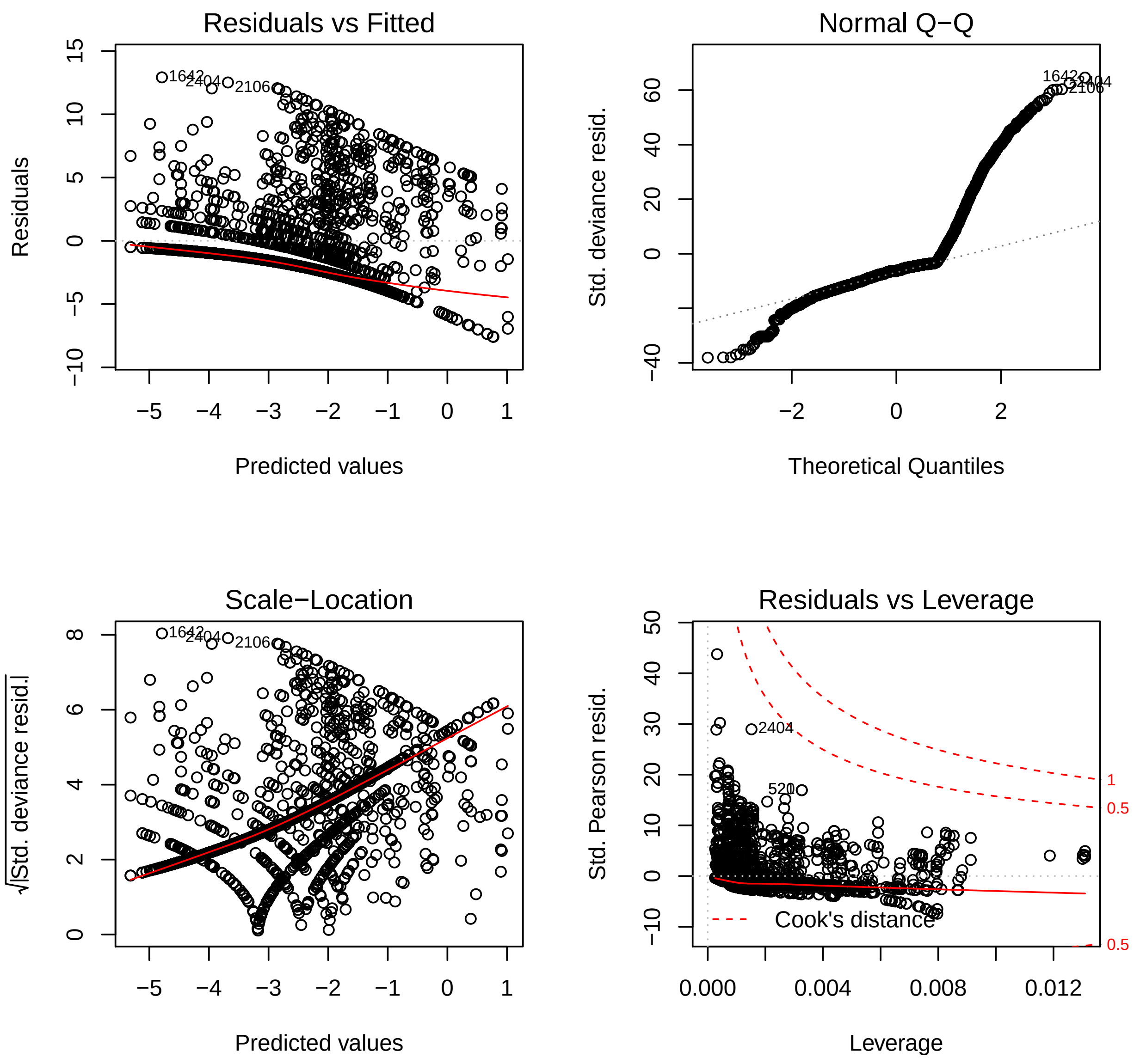

Supplement: Supplementary file 1 [file ECE3-7-4640-s001.pdf]
